# Thermal noise in aqueous quadrupole micro- and nano-traps

**DOI:** 10.1186/1556-276X-7-156

**Published:** 2012-02-27

**Authors:** Jae Hyun Park, Predrag S Krstić

**Affiliations:** 1 Physics Division, Oak Ridge National Laboratory, Oak Ridge, TN, 37831, USA

**Keywords:** aqueous quadrupole trap, Brownian motion, random thermal noise, rms fluctuation, charged bio-molecule, DNA sequencing

## Abstract

Recent simulations and experiments with aqueous quadrupole micro-traps have confirmed a possibility for control and localization of motion of a charged particle in a water environment, also predicting a possibility of further reduction of the trap size to tens of nano-meters for trapping charged bio-molecules and DNA segments. We study the random thermal noise due to Brownian motion in water which significantly influences the trapping of particles in an aqueous environment. We derive the exact, closed-form expressions for the thermal fluctuations of position and velocity of a trapped particle and thoroughly examine the properties of the rms for the fluctuations as functions of the system parameters and time. The instantaneous signal transferring mechanism between the velocity and position fluctuations could not be achieved in the previous phase-average approaches.

## Introduction

Conventional quadrupole Paul traps [1,2] are used to confine the charged particles (e.g., atomic and molecular ions) to narrow three-dimensional (3-D) or two-dimensional (2-D) regions by the combination of static (DC) and radio-frequency (rf, AC) oscillating electric fields in vacuum or in gaseous environment. Their applications include mass spectrometry [3], quantum information processing [4,5], micro-dynamical sensors [6], etc. While 3-D trap confines the charged particles to the trap center, the 2-D (so-called linear) Paul trap confines the particles to the trap axis.

The aqueous Paul nano-trap (APT) is a quadrupole trapping device for the confinement of nano-sized objects in water (and possible electrolyte) using rf electric field. Recent theoretical [7-10] and experimental [11,12] studies show feasibility of the aqueous Paul traps for localization and control of the motion of charged micro- and nano-particles. Presence of aqueous and possible electrolytic [10,11] environment is of the key importance for chemical stability of charged bio-molecules. In particular, control of translocation of a single-stranded DNA by APT may improve the performance of the third generation of DNA sequencing devices through synthetic nano-pores [13,14]. A bio-molecule translocation application determines our interest to a linear Paul trap. The influence of the thermal fluctuations in the dense water environment to the linear (2-D) Paul micro- and nano-trap functions is the main focus of this paper.

An important factor in designing an aqueous Paul trap is its stability characteristics, i.e., range of the system parameters for which a targeted charged particle stays a sufficiently long time in a confinement region to provide the desired functions and manipulations. The stability of a spherical-charged particle in a conventional Paul trap in vacuum or in a buffer gas at a low pressure is mainly determined by the dimensionless '*a*' and '*q*' parameters

$$a=\frac{2QU}{M\Omega^{2}r_{0}^{2}}$$

$$q=\frac{2QV}{M\Omega^{2}r_{0}^{2}},$$

where *M *and *Q *are the mass and charge of the particle, respectively; *U *and *V *are the DC and AC voltages, respectively; *Ω *is the frequency of the AC input; and *r_0 _*is the radius of the Paul trap [15]. Diameter of the particle is assumed to be much smaller than the trap radius. The stability of an aqueous Paul trap is also influenced by the viscous drag of the surrounding water expressed by the parameter '*b*'

$$b=\frac{2\xi }{M\Omega },$$

where *ξ *is the friction coefficient of a non-slip spherical particle in Stokes' drag, *ξ *= 6*πηa*_p_, *η *is the viscosity of medium and *a*_p _is the radius of the particle. According to the fluctuation-dissipation theorem [16], the magnitude of the random force is proportional to *k*_B_*Tξ *[17,18] where *k*_B _is the Boltzmann constant and *T *is the liquid temperature. The viscosity of water (8.9 × 10^-4 ^Pa·s) is about 50 times larger than of air (1.78 × 10^-5 ^Pa·s at *T *= 298 K), i.e., a particle in water experiences about 50 times larger random force than in the air. Therefore, understanding the functions of the Paul trap filled with water (or more general, with a high viscous medium) requires, in addition to the stability analysis based on the mean motion of particle, also a detailed understanding of its response to the thermal fluctuations. Although the mean motion may be stable, i.e., converging to the trap center [10-12], a presence of large thermal fluctuations of the particle may suppress or even prevent its localization and control.

The fluctuations of a charged micro-particle in gaseous quadrupole Paul trap have been studied intensively in the past. Arnolds et al. numerically computed the fluctuation of position by using Langevin equation [19] and Fokker-Planck equation [20]. They found that the numerical results are in a good agreement with their experimental data in air at atmospheric pressure in tens of Hz range of the applied AC frequencies. Thus, they trapped a few micrometer-sized particles in a millimeter-sized Paul trap (2*r_0 _*= 9 mm) using an AC electric bias of *V *= 1.0 V and *Ω *= 60 Hz, resulting in less than 1.0 μm fluctuations. Blatt et al. [21] and Zerbe et al. [22] computed the thermal fluctuations of position and velocity by using Fokker-Planck equation in a gas medium in the limit of small b-parameters. Joos and Lindner [23] derived the series expansions of the thermal fluctuations of position and velocity from the Langevin equation in the limit of small *q *parameters.

In the present study, we solve the relevant Langevin equation in a closed form analytically in terms of integrals of Mathieu functions [24,25]. The derived formulas are quite general and applicable to arbitrary range of trap parameters of an aqueous quadrupole trap, enabling us to fully analyze the transient behavior of the thermal fluctuations, their power spectrum density (PSD), position and velocity fluctuations, as well as their covariance.

We consider the linear (2-D) aqueous quadrupole Paul trap because many interesting bio-molecules (e.g., DNA, RNA) are long-charged polymers that could be translocated along the trap axis with localization in the trap center. A generalization to the 3-D Paul trap is obvious and straightforward, and will not be pursued here.

**Figure 1:**
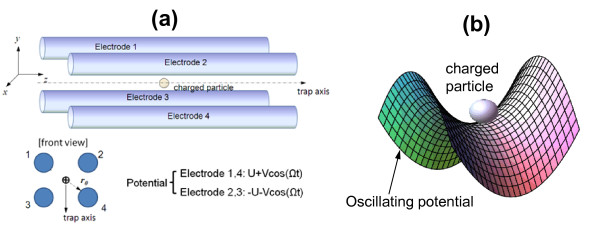
**Overview of quadrupole trapping of charged particle**. (**a**) Schematics of a linear aqueous quadrupole trap. At electrode 1 and 4, the potential is *U *+ *V*cos(*Ωt*), while on electrode 2 and 3, the potential is -*U *- *V*cos(*Ωt*). *r*_0 _is the trap radius, and *Ω *is the driving angular frequency of AC input. (**b**) The charged particle is confined around the trap axis.

As indicated in Figure 1, a point-like spherical charged particle in a 2-D quadrupole Paul trap experiences the oscillating electric potential, in addition to viscous damping, and the stochastic random force due to the random collisions with the molecules of the viscous medium. The Langevin-type equation of motion (EOM) has this form:

(1) $$M\frac{d^{2}r}{d\;t^{2}}=-\xi \frac{d\;r}{d\;t}+Q\left(-\nabla \Phi \right)+R\left(t\right)$$

where *t *is time and **r **is the position vector of particle, $r=x{\hat{e}}_{x}+y{\hat{e}}_{y}$. The three terms on the right hand side (RHS) of Equation 1 are the damping force, the electrophoretic force due to the gradient of the electric potential *Φ*, and the Brownian random force, respectively. Since the AC-only case (i.e., when the DC voltage *U *in definition of the *a *parameter is equal to zero) can provide a considerable stable region [26], we focus in this study to that case without loss of generality. Since the EOMs in x and y directions differ mutually only by sign of the electrophoretic force [7,8,25], it is sufficient to solve the EOM in one direction (x for example):

(2) $$M\frac{d\;x}{d\;t}+\xi \frac{d\;x}{d\;t}-Q\frac{V\cos\Omega t}{r_{0}^{2}}x=R\left(t\right).$$

For brevity, *R*(*t*) here is the random force component in the x direction. When *R*(*t*) = 0, Equation 2 can be reduced, using the transformation $x\left(t\right)=e^{-\frac{b}{2}t}p\left(t\right)$, to the Mathieu differential equation, leading to Mathieu functions.

The random force [16-18] vanishes in the mean,

(3a) $$\left\langle R\left(t\right)\right\rangle =0,$$

is uncorrelated with the velocity *v*(*t*) at any earlier time,

(3b) $$\left\langle v\left(t\right)R\left(t^{\prime }\right)\right\rangle =0\;\;t^{\prime }>t,$$

and its correlation time is infinitely short, namely the autocorrelation function of *R*(*t*) has the form:

(3c) $$\left\langle R\left(t\right)R\left(t^{\prime }\right)\right\rangle =G_{R}\delta \left(t-t^{\prime }\right)$$

where < > means the statistical average over an ensemble of particles. *G*_R _is the constant spectral density in power spectrum of the random force. Using fluctuation-dissipation theorem, Kubo [16] showed that *G*_R _is related to the environment friction coefficient by Equation 4:

(4) $$G_{\text{R}}=2k_{B}T\xi$$

The random force satisfying the *δ*-function correlation of Equation 3c is called 'white-noise' [17].

In addition to the charge-dependent electrophoretic force, *Q*(-∇Φ), a particle in an aqueous environment and in non-uniform electric field could experience the dielectrophoretic (DEP) force due to the difference between dielectric constants and conductivities between particle and the environment. Our analysis showed [10,26] that the effects of dielectrophoretic force becomes dominant for small values of *q *(<< 1) (and *a*) parameters. However, when *q *> 0.01, the stability of particle is dominated by the electrophoretic force [26]. The Brownian motion including DEP forces is discussed elsewhere [10].

## Theoretical analysis

In this section, the explicit closed-form analytical expressions for thermal fluctuations of position and velocity, and the cross-covariance of position and velocity are derived in terms of integrals of Mathieu functions by solving the equation of motion in Langevin form for a charge particle in an aqueous quadrupole Paul trap.

### Thermal fluctuations of the position

The Equation 3b can be re-written in the following form:

(5) $$\frac{d^{2}x_{1}}{dt_{1}^{2}}+b\frac{dx_{1}}{dt_{1}}-2q\cos\left(2t_{1}\right)x_{1}=\frac{2}{\Omega }R\left(\frac{2t_{1}}{\Omega }\right),$$

where we introduce the scaled variables

(6) $$t_{1}=\frac{\Omega }{2}t,\;x_{1}=\frac{M\Omega }{2}x$$

and use the dimensionless parameters *b *and *q*, defined in the 'Introduction' section. Assuming the initial conditions *x*_1_(*t*_1 _= 0) = *x*_10 _and ${\left(\frac{d\;x_{1}}{d\;t_{1}}\right|}_{t_{1}=0}=v_{10}$, the closed form analytical solution of Equation 2 is obtained in the form below:

(7) $$\begin{array}{c} x_{1}\left(t_{1}\right)=x_{10}e^{-\frac{1}{2}bt_{1}}c\left(-\frac{1}{4}b^{2},q,t_{1}\right)+\left(v_{\text{x}10}+\frac{1}{2}bx_{0}\right)e^{-\frac{1}{2}bt_{1}}s\left(-\frac{1}{4}b^{2},q,t_{1}\right)\\ \;\;\;-\int_{0}^{t_{1}}s\left(-\frac{1}{4}b^{2},q,u\right)c\left(-\frac{1}{4}b^{2},q,t_{1}\right)\left[\frac{2}{\Omega }\cdot R\left(\frac{2}{\Omega }u\right)\right]e^{\frac{1}{2}b\left(u-t_{1}\right)}du\\ \;\;\;+\int_{0}^{t_{1}}c\left(-\frac{1}{4}b^{2},q,u\right)s\left(-\frac{1}{4}b^{2},q,t_{1}\right)\left[\frac{2}{\Omega }\cdot R\left(\frac{2}{\Omega }u\right)\right]e^{\frac{1}{2}b\left(u-t_{1}\right)}du, \end{array}$$

where *c*(*a*,*q*,*t*) and *s*(*a*,*q*,*t*) are the Mathieu cosine and sine functions, respectively. Hereafter, we use the notation *a *= -b^2 ^/4. At the RHS of Equation 7, the first two terms express the instantaneous motion of a particle in the Paul trap without influence of random force, while the rest of the equation is due to the thermal fluctuations, i.e., due to the random force *R*(*t*). The property of Mathieu functions, *c*(*a*,*q*,*t*)*s*'(*a*,*q*,*t*)-*s*(*a*,*q*,t)*c*'(*a*,*q*,*t*) = 1 [27], is used in derivation of Equation 7.

**Figure 2:**
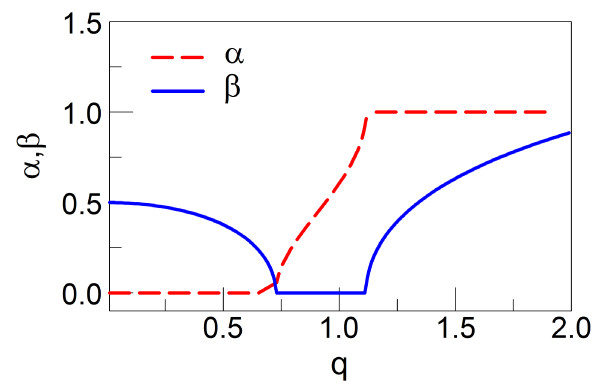
**Variation of Mathieu exponent for *b *= 1**. Variation of Mathieu exponent with *q *for *b *= 1. *α *and *β *are the real and imaginary parts of Mathieu exponent *μ*, respectively.

Applying the relations for white noise given in Equations 3c and 4, the square of fluctuations of position follows in the form:

(8) $$\sigma_{\text{xx}}=\left\langle {\left[x-\left\langle x\right\rangle \right]}^{2}\right\rangle =2b\cdot {\left(\frac{2v_{\text{eq}}}{\Omega }\right)}^{2}I\left(b,q,t_{1}\right),$$

where *I*(*b*,*q*,*t*_1_) is the integral defined by Equation 15 in Appendix 1 and *v*_eq _is the equilibrium velocity given by equipartition theorem as$\sqrt{\frac{k_{B}T}{M}}$[28]. We note that there is no overlap between random and mean motion in Paul trap with white random noise. In order that *I*(*b*,*q*,*t*_1_) is physically meaningful, it has to be bounded. The mean motion of particle in a quadrupole Paul trap is stable for *β *<*b */2 where *β *is the imaginary part of Mathieu exponent, *μ *= *α *+ *iβ*. This is exactly the condition for stability in the trap, i.e., non-divergence of *I*(*b*,*q*,*t*_1_) [29]. As long as the particle is in the stable region, the fluctuations have the finite values. However, if the charged particle becomes unstable, both mean motion and fluctuations diverge simultaneously. By using the condition 0 <*β *<*b */2, one can compute the stability borders for the aqueous Paul trap. For example, in Figure 2 with *b *= 1.0, the condition of 0 <*β *<*b */2 = 0.5 is satisfied with 0 <*q *< 1.3, which defines the boundary of the stable region. Other *b *values are described in Figure S1 [see Additional file 1]. In the limit *q*→ 0, i.e., in the absence of the external AC electric field, Equation 8 is reduced to the mean square displacement (MSD) relation for diffusion in the long-time limit, *σ*_xx _= 2*Dt*, where *D *is diffusion coefficient given as Einstein's relation $\left(D=\frac{k_{B}T}{\xi }\right)$[30]. For *b*→ 0, i.e., when the viscous drag is negligible, *σ*_xx_→ 0. This is consistent with the fact that the fluctuations disappear in a non-viscous environment [16].

### Thermal fluctuations of velocity

The expression for thermal fluctuations of velocity is derived by the time differentiation of Equation 7 and the application of the Gaussian random force properties of Equation 3 as was done for the fluctuations of the position. The explicit expression for velocity is presented in Appendix 2. The final expression of the square of the fluctuations of the velocity is obtained in the form:

(9) $$\sigma_{\text{vv}}=\left\langle {\left[v-\left\langle v\right\rangle \right]}^{2}\right\rangle =2b\cdot v_{\text{eq}}^{2}J\left(b,q,t_{1}\right)$$

When *q*→ 0, $\sigma_{\text{vv}}\to v_{\text{eq}}^{2}$at long-time limit. For *b*→ 0, the velocity fluctuations vanish as the position fluctuations do.

### Covariance of position and velocity fluctuations

The covariance of position and velocity fluctuations, which describes the correlation of these two quantities, is computed by multiplying the position expression of Equation 7 and the velocity expression of Equation 20, and taking ensemble average with random force properties from Equations 3. The final expression for the covariance follows in the form:

(10) $$\sigma_{\text{xv}}=\left\langle \left[x-\left\langle x\right\rangle \right]\left[v-\left\langle v\right\rangle \right]\right\rangle =2b\cdot \left(\frac{2v_{\text{eq}}^{2}}{\Omega }\right)K\left(b,q,t_{1}\right),$$

where the *K *function is defined in Appendix 3. The influence of the covariance to the fluctuations will be discussed in the next section.

## Discussion

**Figure 3:**
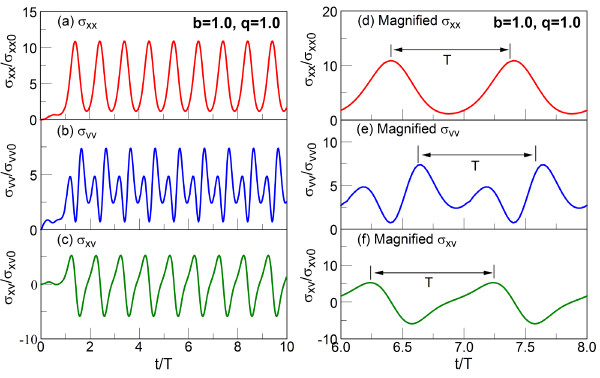
**Transient behaviour of thermal noises**. Time dependent behaviors of *σ*_xx _(red), *σ*_vv _(blue), and *σ*_xv _(green) for *b *= 1.0 and *q *= 1.0 (curves a to c). *T *is the period corresponding to the driving frequency of *T *= 2*π */*Ω*. *σ*_xx0_, *σ*_vv0_, and *σ*_xv0 _are defined as (2*v*_eq_/Ω)^2^,$v_{\text{eq}}^{2}$, and $2v_{\text{eq}}^{2}/)\Omega$, respectively. Magnified figure for 6*T *<*t *< 8*T *(curves d to f).

As shown in Appendices 1, 2 and 3, *σ*_xx_, *σ*_vv_, and *σ*_xv _can be expressed in terms of integrals *J*_1_(*b*,*q*,*t*_1_), *J*_2_(*b*,*q*,*t*_1_), and *J*_3_(*b*,*q*,*t*_1_). On the other hand, computations of *J*_1_(*b*,*q*,*t*_1_), *J*_2_(*b*,*q*,*t*_1_), and *J*_3_(*b*,*q*,*t*_1_) are straightforward due to the periodic property of Floquet solution, as shown in Appendix 1. Figure 3 shows the temporal histories of *σ*_xx_, *σ*_vv_, and *σ*_xv _for *b *= 1.0 and *q *= 1.0. We note that these values of *b *and *q *correspond to the minimum position fluctuations in the long-time limit, as will be shown later. In the figure, the time at x-axis is normalized by the period of driving excitation, *T *= 2*π */*Ω*. The Mathieu exponent for the parameters *b *= 1.0, *q *= 1.0 is *μ *= 0.6252 + *i*0, and the particle is in the stable region. We set *σ*_xx_, *σ*_vv_, and *σ*_xv _to zero at the initial time, *t *= 0. These gradually increase for *t *≤ *T*, and the curves show a periodic behavior, which becomes a steady oscillation, i.e., a particle oscillation amplitude does not change (with the numerical error of 10^-4^) after a few cycles. Thus, the particle dynamics reaches a steady oscillatory state in the 'long-time limit'. In the curves a, b, and c of Figure 3, the long-time limit is reached after approximately 6 *T*, with the same period of oscillations, *T*, for *σ*_xx_, *σ*_vv_, and *σ*_xv_, as seen in the curves d, e, and f of Figure 3. However, the detailed features of the *σ*_xx_, *σ*_vv_, and *σ*_xv _are different in the long-time limit (superscript ∞). Thus, $\sigma_{\text{xx}}^{\infty }$ and $\sigma_{\text{vv}}^{\infty }$ are oscillating around a non-zero value. The previous theoretical studies have focused mostly on the phase-averaged values in long-time limit [19,20] where it has been difficult to establish a physical relationship between position and velocity fluctuations since the average covariance between them is zero as seen in curve f of Figure 3.

Near the *q *value, for which the position fluctuation becomes minimum, for example, *q *= 1.5 for *b *= 2.0 (Mathieu exponent is *μ *= 0 + 0.3687*i*) and *q *= 3.1 for *b *= 4.0 (*μ *= 0 + 1.6262*i*), the long-time behaviors are not much different except for the reduction of amplitude of the position fluctuations. For (*b*,*q*) = (2.0, 1.5) and (4.0, 3.1) as well as for (1.0, 1.0), the Mathieu exponents do not have any real part, and all three fluctuations, $\sigma_{\text{xx}}^{\infty }$, $\sigma_{\text{vv}}^{\infty }$, and $\sigma_{\text{xv}}^{\infty }$, oscillate in phase with angular frequency *Ω *= 2*π */*T*.

The embedded frequencies in $\sigma_{\text{xx}}^{\infty }$, $\sigma_{\text{vv}}^{\infty }$, and $\sigma_{\text{xv}}^{\infty }$ can be analyzed by investigating their PSDs which are computed as the absolute values of Fourier transform of the fluctuations, shown in Equation 11:

(11) $$S_{ij}^{\infty }\left(\omega \right)={\left|\int_{-\infty }^{+\infty }\left[\frac{\sigma_{pq}^{\infty }\left(t\right)}{\sigma_{pq0}}\right]e^{-j\omega t}dt\right|}^{2}\;\;\left(p,q=x,v\right).$$

**Figure 4:**
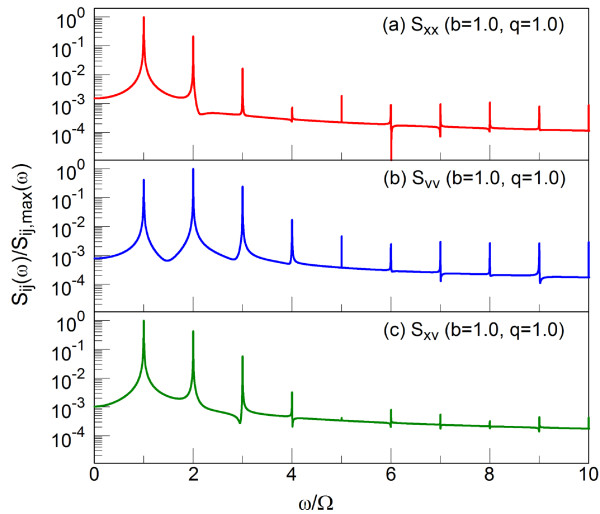
**Power spectrum densities for *b *= 1.0 and *q *= 1.0**. Power spectrum density of position fluctuation *σ*_xx _(red), velocity fluctuation *σ*_vv _(blue), and covariance *σ*_xv _(green).

Figure 4 reveals the PSDs for $\sigma_{\text{xx}}^{\infty }$, $\sigma_{\text{vv}}^{\infty }$ and $\sigma_{\text{xv}}^{\infty }$ in logarithmic scale for *b *= 1.0 and *q *= 1.0. The frequency at x-axis is normalized by the driving frequency *Ω*, and the maximum of PSD is normalized to one. The peaks in PSD appear at every *Ω *period, revealing the presence of the high harmonics whose amplitudes decrease fast at the higher harmonics whose order is larger than 3. For $\sigma_{\text{xx}}^{\infty }$ and $\sigma_{\text{xv}}^{\infty }$, the peak at *ω *= *Ω *is dominant, while for $\sigma_{\text{vv}}^{\infty }$, the second peak at *ω *= 2*Ω *slightly dominates over the one at *ω *= *Ω *and *ω *= 3*Ω*. This observation is consistent with the one in Figure 3 (curves d, e, and f). Thus, both $\sigma_{\text{xx}}^{\infty }$ and $\sigma_{\text{xv}}^{\infty }$ have a quite regular oscillation with period *T*, while $\sigma_{\text{vv}}^{\infty }$ shows a more complex time evolution.

Since $\sigma_{\text{xx}}^{\infty }$, $\sigma_{\text{vv}}^{\infty }$, and $\sigma_{\text{xv}}^{\infty }$ are oscillating with the driving frequency *Ω *(and higher harmonics of frequencies *nΩ*), one can define their mean values by time-averaging over *T*

(12a) $${\bar{\sigma }}_{\text{xx}}^{\infty }=\sigma_{\text{xx}0}\cdot 2b\cdot {\bar{J}}_{3}^{\infty }\left(b,q\right),$$

(12b) $${\bar{\sigma }}_{\text{vv}}^{\infty }=\sigma_{vv0}\cdot 2b\cdot \left[{\bar{J}}_{1}^{\infty }\left(b,q\right)-b{\bar{J}}_{2}^{\infty }\left(b,q\right)+\frac{b^{2}}{4}{\bar{J}}_{3}^{\infty }\left(b,q\right)\right],$$

(12c) $${\bar{\sigma }}_{\text{xv}}^{\infty }=\sigma_{\text{xv}0}\cdot 2b\cdot \left[{\bar{J}}_{2}^{\infty }\left(b,q\right)\text{ - }\frac{b}{2}{\bar{J}}_{3}^{\infty }\left(b,q\right)\right],$$

with

(12d) $$\sigma_{\text{xx}0}={\left(\frac{2v_{\text{eq}}}{\Omega }\right)}^{2},$$

(12e) $$\sigma_{\text{vv}0}=v_{\text{eq}}^{2},$$

(12f) $$\sigma_{\text{xv}0}=\frac{2v_{\text{eq}}^{2}}{\Omega },$$

and

(12g) $${\bar{J}}_{i}^{\infty }\left(b,q\right)=\frac{1}{T}\int_{0}^{T}J_{i}^{\infty }\left(b,q,\tau \right)\;d\tau \;\left(i=1,2,3\right).$$

It is obvious that $\sigma_{\text{vv}0}=v_{\text{eq}}^{2}$ expresses the thermal equilibrium velocity of molecules. In the aqueous Paul trap with driving frequency *Ω*, the characteristic time is *t*_c _= *T *= 2*π */*Ω*. So, $\sqrt{\sigma_{\text{xx}0}}=\frac{2v_{\text{eq}}}{\Omega }$ is the characteristic length (*L*_c_) for random motion in Paul trap. Also, $\sigma_{\text{xv}0}=\frac{2v_{\text{eq}}^{2}}{\Omega }$ has the dimension of and can be interpreted as the effective diffusion coefficient (*D*_eff,*PT*_) for the signal diffusion due to the random motion during a cycle of the external periodic driving excitation. Interestingly, the following relation holds among the pre-factors *σ*_xv0 _and *σ*_xx0_:

(13) $$\sigma_{\text{xv}0}=2\sigma_{\text{xv}0}\cdot t_{\text{c}}\Rightarrow L_{\text{c}}^{2}=2D_{\text{eff},\text{PT}}\cdot t_{\text{c}},$$

This is exactly the same as the MSD relation for diffusion in absence of the driving field.

**Figure 5:**
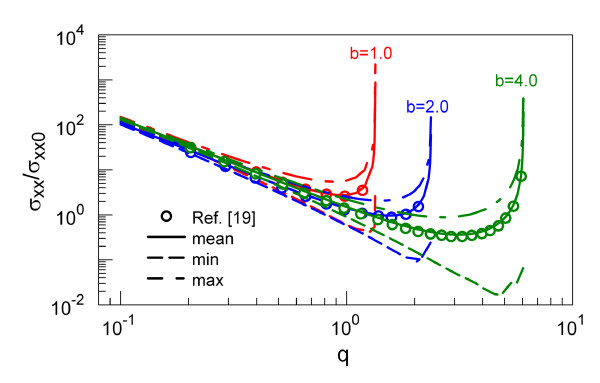
**Mean thermal noise of position**. Variations of ${\bar{\sigma }}_{\text{xx}}^{\infty }/)\sigma_{\text{xx}0}$ with *q *for different *b*.

In Figure 5, the scaled variation of ${\bar{\sigma }}_{\text{xx}}^{\infty }/)\sigma_{\text{xx}0}$ with *q *at a given *b *is presented. The mean value of ${\bar{\sigma }}_{\text{xx}}^{\infty }$ agrees very well with the results from Arnold et al. [19] which solved Equation 2 numerically using the Green's function random-phase approach. The *q *value in which ${\bar{\sigma }}_{\text{xx}}^{\infty }/)\sigma_{\text{xx}0}$ becomes unbounded (i.e., unstable) increases with *b*, and this defines the stability border. At the border, both $\sigma_{\text{xx},\max}^{\infty }$ and $\sigma_{\text{xx},\min}^{\infty }$ diverge.

As we discussed above, both mean trajectory and its thermal fluctuation amplitude diverge simultaneously in unstable region. The influence of random force to the stability border is negligible. The mean value of ${\bar{\sigma }}_{\text{xx}}^{\infty }$ has, for all *b*s, a local minimum (*q*_min,xx_) in the stable region, close to the stability border. The *q*_min,xx _increases with the increase of *b*. Near stability border, the ratio of maximum to minimum trajectory fluctuation $\sigma_{\text{xx},\max}^{\infty }/)\sigma_{\text{xx},\min}^{\infty }$ becomes large (approximately 10) for all *b*s. The temporal histories of *σ*_xx_, *σ*_vv_, and *σ*_xv _for larger *b *= 4.0 are presented in Figure S2 [see Additional file 1]. With larger *b*, the minimum values of *σ*_xx _and *σ*_vv _are significantly reduced which corresponds to the reduction of minimum *σ*_xx _with increase of *b *in Figure 5.

**Figure 6:**
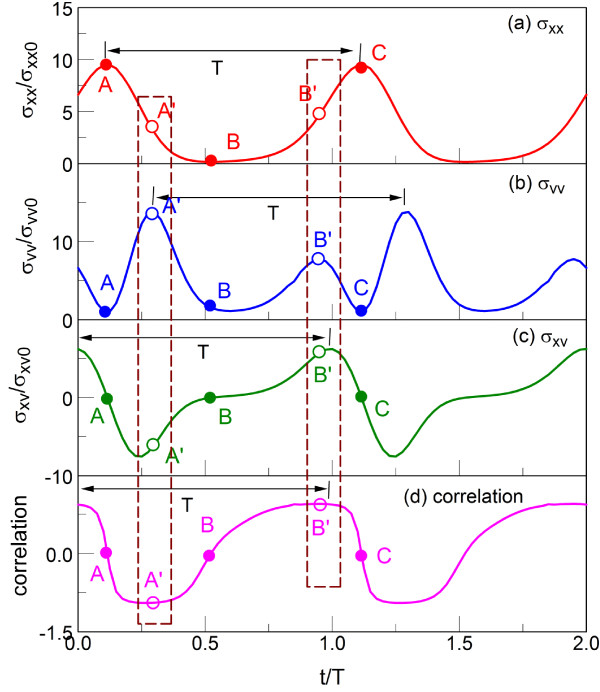
**Detailed understanding of thermal noises for *b *= 4.0 and *q *= 4.0**. Enlarged view of transient behaviors of (a) *σ*_xx_, (b) *σ*_vv_, (c) *σ*_xv_, and (d) correlation.

In Figure 6, we examine *σ*_xx_, *σ*_vv_, *σ*_xv_, and the correlation between position and velocity fluctuations for an arbitrary 2 *T *interval in the long-time limit for *b *= 4.0 and *q *= 4.0. We can compute the correlation as a scaled version of covariance (*σ*_xv_) representing the degree of similarity of two random variables, defined as

(14) $$correlation\left\{x,v\right\}=\frac{\sigma_{\text{xv}}}{\sqrt{\sigma_{\text{xx}}}\sqrt{\sigma_{\text{vv}}}}.$$

The correlation varies between -1 to 1 since the covariance can be both positive and negative. The variables are positively and negatively correlated for the positive and negative correlation, respectively. Variables *x *and *v *could be uncorrelated when correlation is 0. Of course, the larger absolute value of the quantity in Equation 14 indicates the stronger correlation between *x *and *v *[31]. It should be averaging of *σ*_xv _over one period (for example, from A to C in curve c of Figure 6) that gives a numerical zero, unlike the correlation, which does not average to zero since it is a non-linear scaling of *σ*_xv _by $\sqrt{\sigma_{\text{vv}}\sigma_{\text{xx}}}$.

In the Figure 6, the zero correlation (covariance) points (A, B, and C) correspond to the minimum *σ*_vv _positions regardless of *σ*_xx_. However, the maximum correlation always occurs when *σ*_vv _has local maximum while *σ*_xx _is near the mean (A' and B'). In other words, when the velocity fluctuations reach its local maximum, the covariance also becomes maximized, and the velocity fluctuation information is easily transferred to the position fluctuation. The fluctuation embedded in the velocity is very sensitive to the variation of the field (for a given *b*). Then, the information propagates to the position fluctuation through the covariance. The covariance acts as a diffusion transfer engine (its physical dimension is diffusion).

**Figure 7:**
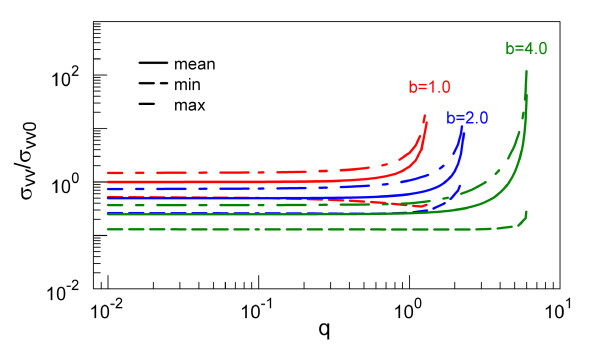
**Mean thermal noises of velocity**. Variations of ${\bar{\sigma }}_{\text{vv}}^{\infty }/)v_{\text{eq}}^{2}$, $\sigma_{\text{vv},\max}^{\infty }/){\bar{\sigma }}_{\text{vv}}^{\infty }$, and $\sigma_{\text{vv},\min}^{\infty }/){\bar{\sigma }}_{\text{vv}}^{\infty }$ with *q *for different *b*.

The detailed features of velocity fluctuations are presented in Figure 7 for *b *= 1.0, *b *= 2.0, and *b *= 4.0. With the increase of *q*, all ${\bar{\sigma }}_{\text{vv}}^{\infty }$, $\sigma_{\text{vv},\max}^{\infty }$, and $\sigma_{\text{vv},\min}^{\infty }$ monotonically increase and finally diverge at the stability border. Also, for *q *< 1.0, the square of ${\bar{\sigma }}_{\text{vv}}^{\infty }$ converges to $2v_{\text{eq}}^{2}$ regardless of the value of *b*, which seems to contradict to the equipartition theorem discussed in the 'Thermal fluctuations of the position' subsection (i.e., *σ*_vv _= $v_{\text{eq}}^{2}$ for *q *= 0). This can be understood in the following way: the limit of *q *= 0 under finite *b *means that the AC voltage is zero, *V *= 0. In this case, the driving frequency becomes meaningless, and we can use *Ω *= 0. If *Ω *= 0, the physical time interval of one period becomes infinite, and by 'averaging' over the first cycle, (0 <*t *<*T *= ∞) we obtain ${\bar{\sigma }}_{\text{vv}}^{\infty }=v_{\text{eq}}^{2}$. The 'radio frequency heating' [19] (*V *> 0) increases the kinetic energy of a trapped particle and consequently ${\bar{\sigma }}_{\text{vv}}^{\infty }=2v_{\text{eq}}^{2}$.

Once we choose the combination of *b *and *q *parameters which provide the stable trap condition, this could be converted into a desired aqueous trap design. Thus, for a polystyrene micro-particle of diameter of 0.8 μm and charge of 10^6 ^Q in a Paul trap of 2*r*_0 _= 8.0 μm [9,10], *b *= 4.26 and *q *= 0.45 correspond to 1.0 V AC at 2 MHz. These parameters then yield the characteristic length of random motion (as defined below Equation 12) of $\sqrt{\sigma_{\text{xx}0}}$ = 0.61 nm, while the thermal velocity of molecules is $\sqrt{\sigma_{\text{vv}0}}$ = 3.85 × 10^-3 ^m/s. The actual rms values of the position fluctuations can be obtained from Figure 5, i.e., $\sqrt{\sigma_{\text{xx}}}$ ≈ 4 nm and $\sqrt{\sigma_{\text{vv}}}$ ≈ 10^-3^m/s (using Figure 7). On the other hand, for a bio-particle radius of 5 nm and a charge of 5 Q, with the driving frequency of 300 MHz and AC voltage of 1.2 V for a trap of *r*_0 _= 40.5 nm, we found that *b *= 1.12 × 10^2 ^and *q *= 0.37 for which the particle is still stable [26]. These yield $\sqrt{\sigma_{\text{xx}0}}$ is approximately 2.3 nm, while the thermal velocity of molecules is $\sqrt{\sigma_{\text{vv}0}}$ approximately 2.2 m/s. These values are beyond the calculated scaling curves in Figures 5 and 7, and the actual rms values in this case have to be obtained by explicit integrations of the Mathieu functions in Equation 12.

## Conclusions

We derive the closed-form analytical expressions for thermal fluctuations of position and velocity of charged particles in aqueous quadruple Paul trap, as well as their covariance starting from Langevin equation with random force. Unlike the conventional Paul trap in vacuum or in air with small random noise, an aqueous Paul trap exhibits relatively large Brownian fluctuations due to the large viscosity in water, depending also on the trap parameters (the trap size, the particle mass and charge, and external electric trapping field amplitude and frequency). The fluctuations are expressed in terms of integrals of the Mathieu cosine and sine functions and their derivatives, applicable for arbitrary values of the dimensionless trap parameters *b *and *q*. In the limiting cases, our results agree well to the values in the literature as well as to the theoretical limits of 'no-external force and 'no-damping'. The thermal fluctuations are still oscillating functions even in the long-time limit. Our approach can be easily extended to the 'colored' noise case [32].

Since our solution is not based on the phase-average approach, we obtain the instantaneous time-dependent coupling between the position and velocity fluctuations. We find that the correlation between position and velocity fluctuations becomes maximized for the maximum velocity fluctuation. Near the unstable region of the trap parameters, the velocity diverges, and this is transferred to the position fluctuation through the covariance. The covariance acts as a diffusion transfer engine. Even though the phase average of covariance is zero, as also indicated in the previous studies [19,20], the covariance itself is not zero at every instant of time, causing strong correlation between position and velocity fluctuations in the aqueous Paul trap.

A big advantage of the aqueous Paul trap is to provide a 'virtual nano-pore' for control of a nano-dimensioned DNA segment, while the actual physical size of the trap could be in the range of tens of nm. This significantly reduces the fabrication effort of the nano-pores as well as the problems of the interaction of the bio-molecule with the material walls.

## Competing interests

The authors declare that they have no competing interests.

## Authors' contributions

JHP and PSK together carried computations, analyzed results, and prepared the manuscript. All authors read and approved the final manuscript.

## Appendices

### Appendix 1. *I*(*b*,*q*,*t*_1_) in Equation 8

*I*(*b*,*q*,*t*_1_) in Equation 8 is expressed as:

(15) $$I\left(b,q,t_{1}\right)=I_{1}\left(b,q,\tau \right)+\frac{1}{4}\left[I_{2a}^{n}\left(b,q,\tau \right)-2I_{2b}^{n}\left(b,q,\tau \right)+I_{2c}^{n}\left(b,q,\tau \right)\right],$$

where the 'time' *t*_1 _= *nπ *+ *τ*, *n *is a non-zero integer and 0 ≤ *τ *<*π *is emerging from the periodicity of Floquet solution of Mathieu equation [23]. The detailed forms of *I*_1_(*b*,*q*,*τ*),$I_{2a}^{n}\left(b,q,\tau \right)$, $I_{2b}^{n}\left(b,q,\tau \right)$, and $I_{2c}^{n}\left(b,q,\tau \right)$ are:

(16) $$I_{1}\left(a,q,\tau \right)=\int_{0}^{\tau }{\left[s\left(a,q,u\right)c\left(a,q,\tau \right)\text{ - }c\left(a,q,u\right)s\left(a,q,\tau \right)\right]}^{2}e^{b\left(u-\tau \right)}du,$$

(17) $$I_{2a}^{n}\left(a,q,\tau \right)=\left(\frac{1-e^{-\left(b+i2\mu \right)n\pi }}{e^{\left(b+i2\mu \right)\pi }-1}\right)\int_{0}^{\pi }e^{b\left(u-\tau \right)}{\left[\frac{F\left(a,q,u\right)F\left(a,q,-\tau \right)}{F\left(a,q,0\right)F^{\prime }\left(a,q,0\right)}\right]}^{2}du,$$

(18) $$I_{2b}^{n}\left(b,q,\tau \right)=\left(\frac{1-e^{-nb\pi }}{e^{b\pi }-1}\right)\int_{0}^{\pi }e^{b\left(u-\tau \right)}\frac{F\left(a,q,u\right)F\left(a,q,-u\right)F\left(a,q,\tau \right)F\left(a,q,-\tau \right)}{{\left[F\left(a,q,0\right)F^{\prime }\left(a,q,0\right)\right]}^{2}}du,$$

(19) $$I_{2c}^{n}\left(b,q,\tau \right)=\left(\frac{1-e^{-\left(b-i2\mu \right)n\pi }}{e^{\left(b-i2\mu \right)\pi }-1}\right)\int_{0}^{\pi }e^{b\left(u-\tau \right)}{\left[\frac{F\left(a,q,-u\right)F\left(a,q,\tau \right)}{F\left(a,q,0\right)F^{\prime }\left(a,q,0\right)}\right]}^{2}du,$$

where *F*(*a*,*q*,*u*) is the Floquet solution of Mathieu function and *a *= -*b*^2^/4.

### Appendix 2. Details of velocity fluctuation, *σ*_vv_

The explicit expression of velocity can be obtained by differentiating the expression for the particle position, Equation 7, with respect to time:

(20) $$\begin{array}{l} v_{1}\left(t_{1}\right)=\frac{d\;x_{1}}{d\;t_{1}}\\ \;=x_{10}\left(c^{\prime }\left(-\frac{1}{4}b^{2},q,t_{1}\right)-\frac{b}{2}c\left(-\frac{1}{4}b^{2},q,t_{1}\right)\right)e^{-\frac{1}{2}bt_{1}}\\ \;\;+\left(v_{x10}+\frac{1}{2}bx_{0}\right)\left(s^{\prime }\left(-\frac{1}{4}b^{2},q,t_{1}\right)-\frac{b}{2}s\left(-\frac{1}{4}b^{2},q,t_{1}\right)\right)e^{-\frac{1}{2}bt_{1}}\\ \;\;-\int_{0}^{t_{1}}\left[s\left(-\frac{1}{4}b^{2},q,u\right)c^{\prime }\left(-\frac{1}{4}b^{2},q,t_{1}\right)-\frac{b}{2}s\left(-\frac{1}{4}b^{2},q,u\right)c\left(-\frac{1}{4}b^{2},q,t_{1}\right)\right]\\ \;\;\;\;\times \left[\frac{2}{\Omega }\cdot R\left(\frac{2}{\Omega }u\right)\right]e^{\frac{1}{2}b\left(u-t_{1}\right)}du\\ \;\;+\int_{0}^{t_{1}}\left[c\left(-\frac{1}{4}b^{2},q,u\right)s^{\prime }\left(-\frac{1}{4}b^{2},q,t_{1}\right)-\frac{b}{2}c\left(-\frac{1}{4}b^{2},q,u\right)s\left(-\frac{1}{4}b^{2},q,t_{1}\right)\right]\\ \;\;\;\;\times \left[\frac{2}{\Omega }\cdot R\left(\frac{2}{\Omega }u\right)\right]e^{\frac{1}{2}b\left(u-t_{1}\right)}du. \end{array}$$

Following the same procedure for *σ*_xx_, *σ*_vv _can be expressed as in Equation 9:

$$\sigma_{\text{vv}}=\left\langle {\left[v-\left\langle v\right\rangle \right]}^{2}\right\rangle =2b\cdot v_{\text{eq}}^{2}J\left(b,q,t_{1}\right)$$

with

(21) $$J\left(b,q,t_{1}\right)=J_{1}\left(b,q,t_{1}\right)-bJ_{2}\left(b,q,t_{1}\right)+\frac{b^{2}}{4}J_{3}\left(b,q,t_{1}\right),$$

where *t*_1 _is dimensionless time. It should be noted that *J*_3_(*b*,*q*,*t*_1_) = *I*(*b*,*q*,*t*_1_), where *I*(*b*,*q*,*t*_1_) defines the square of the position fluctuations. Therefore, the square of velocity fluctuations is defined by the sum of a term proportional to the position fluctuations and two more quantities, *J*_1_(*b*,*q*,*t*_1_) and *J*_2_(*b*,*q*,*t*_1_).

Expression for *J*_1_(*b*,*q*,*t*_1_) is

(22) $$J_{1}\left(a,q,t_{1}\right)=J_{11}\left(a,q,\tau \right)+\frac{1}{4}\left[J_{12a}^{n}\left(a,q,\tau \right)+2J_{12b}^{n}\left(a,q,\tau \right)+J_{12c}^{n}\left(a,q,\tau \right)\right],$$

with

(23) $$J_{11}\left(a,q,\tau \right)=\int_{0}^{\tau }{\left[s\left(a,q,u\right)c^{\prime }\left(a,q,\tau \right)-c\left(a,q,u\right)s^{\prime }\left(a,q,\tau \right)\right]}^{2}e^{b\left(u-\tau \right)}du,$$

(24) $$J_{12a}^{n}\left(a,q,\tau \right)=\frac{\left(1-e^{-\left(b+i2\mu \right)n\pi }\right)}{e^{\left(b+i2\mu \right)\pi }-1}\int_{0}^{\pi }e^{b\left(v-\tau \right)}{\left[\frac{F\left(a,q,u\right)F^{\prime }\left(a,q,-\tau \right)}{F\left(a,q,0\right)F^{\prime }\left(a,q,0\right)}\right]}^{2}du,$$

(25) $$J_{12b}^{n}\left(a,q,\tau \right)=\frac{\left(1-e^{-nb\pi }\right)}{e^{b\pi }-1}\int_{0}^{\pi }e^{b\left(v-\tau \right)}\frac{F\left(a,q,u\right)F^{\prime }\left(a,q,-\tau \right)F\left(a,q,\tau \right)F^{\prime }\left(a,q,-u\right)}{{\left[F\left(a,q,0\right)F^{\prime }\left(a,q,0\right)\right]}^{2}}du,$$

(26) $$J_{12c}^{n}\left(a,q,\tau \right)=\frac{\left(1-e^{-\left(b-i2\mu \right)n\pi }\right)}{e^{\left(b+i2\mu \right)\pi }-1}\int_{0}^{\pi }e^{b\left(v-\tau \right)}{\left[\frac{F\left(a,q,-u\right)F^{\prime }\left(a,q,\tau \right)}{F\left(a,q,0\right)F^{\prime }\left(a,q,0\right)}\right]}^{2}du.$$

Expression for *J*_2_(*b*,*q*,*t*_1_) is

(27) $$J_{2}\left(a,q,t_{1}\right)=J_{21}\left(a,q,\tau \right)+\frac{1}{4}\left[J_{22a}^{n}\left(a,q,\tau \right)+J_{22b}^{n}\left(a,q,\tau \right)-J_{22c}^{n}\left(a,q,\tau \right)\right],$$

with

(28) $$\begin{array}{c} J_{21}\left(a,q,\tau \right)=\int_{0}^{\tau }\left\{\left[s\left(a,q,u\right)c^{\prime }\left(a,q,\tau \right)-c\left(a,q,u\right)s^{\prime }\left(a,q,\tau \right)\right]\right)\\ \;\;\;\times \left(\left[s\left(a,q,u\right)c\left(a,q,\tau \right)-c\left(a,q,u\right)s\left(a,q,\tau \right)\right]\right\}e^{b\left(u-\tau \right)}du, \end{array}$$

(29) $$J_{22a}^{n}\left(a,q,\tau \right)=\left(\frac{1-e^{-\left(b+i2\mu \right)n\pi }}{e^{\left(b+i2\mu \right)\pi }-1}\right)\int_{0}^{\pi }e^{b\left(v-\tau \right)}\frac{{\left[F\left(a,q,u\right)\right]}^{2}F\left(a,q,-\tau \right)F^{\prime }\left(a,q,-\tau \right)}{F\left(a,q,0\right)F^{\prime }\left(a,q,0\right)}du,$$

(30) $$\begin{array}{c} J_{22b}^{n}\left(a,q,\tau \right)=\left(\frac{1-e^{-nb\pi }}{e^{b\pi }-1}\right)\\ \;\;\;\times \int_{0}^{\pi }e^{b\left(u-\tau \right)}\frac{F\left(a,q,u\right)F\left(a,q,-u\right)\left[F^{\prime }\left(a,q,\tau \right)F\left(a,q,-\tau \right)+F\left(a,q,\tau \right)F^{\prime }\left(a,q,-\tau \right)\right]}{{\left[F\left(a,q,0\right)F^{\prime }\left(a,q,0\right)\right]}^{2}}du, \end{array}$$

(31) $$J_{22c}^{n}\left(a,q,\tau \right)=\left(\frac{1-e^{-\left(b-i2\mu \right)n\pi }}{e^{\left(b+i2\mu \right)\pi }-1}\right)\int_{0}^{\pi }e^{b\left(v-\tau \right)}\frac{{\left[F\left(a,q,-u\right)\right]}^{2}F\left(a,q,\tau \right)F^{\prime }\left(a,q,\tau \right)}{{\left[F\left(a,q,0\right)F^{\prime }\left(a,q,0\right)\right]}^{2}}du.$$

Finally, expression for *J*_3_(*b*,*q*,*t*_1_) is

(32) $$J_{3}\left(a,q,t_{1}\right)=J_{31}\left(a,q,\tau \right)+\frac{1}{4}\left[J_{32a}^{n}\left(a,q,\tau \right)-2J_{32b}^{n}\left(a,q,\tau \right)+J_{32c}^{n}\left(a,q,\tau \right)\right],$$

with

(33) $$J_{31}\left(a,q,\tau \right)=\int_{0}^{\tau }{\left[s\left(a,q,u\right)c\left(a,q,\tau \right)-c\left(a,q,u\right)s\left(a,q,\tau \right)\right]}^{2}e^{b\left(u-\tau \right)}du,$$

(34) $$J_{32a}^{n}\left(a,q,\tau \right)=\left(\frac{1-e^{-\left(b+i2\mu \right)n\pi }}{e^{\left(b+i2\mu \right)\pi }-1}\right)\int_{0}^{\pi }e^{b\left(u-\tau \right)}{\left[\frac{F\left(a,q,u\right)F\left(a,q,-\tau \right)}{F\left(a,q,0\right)F^{\prime }\left(a,q,0\right)}\right]}^{2}du,$$

(35) $$J_{32b}^{n}\left(a,q,\tau \right)=\left(\frac{1-e^{-nb\pi }}{e^{b\pi }-1}\right)\int_{0}^{\pi }e^{b\left(u-\tau \right)}\frac{F\left(a,q,u\right)F\left(a,q,-u\right)F\left(a,q,\tau \right)F\left(a,q,-\tau \right)}{{\left[F\left(a,q,0\right)F^{\prime }\left(a,q,0\right)\right]}^{2}}du,$$

(36) $$J_{32c}^{n}\left(a,q,\tau \right)=\left(\frac{1-e^{-\left(b-i2\mu \right)n\pi }}{e^{\left(b-i2\mu \right)\pi }-1}\right)\int_{0}^{\pi }e^{b\left(u-\tau \right)}{\left[\frac{F\left(a,q,-u\right)F\left(a,q,\tau \right)}{F\left(a,q,0\right)F^{\prime }\left(a,q,0\right)}\right]}^{2}du.$$

### Appendix 3. Details of covariance, *σ*_xv_

The explicit expression of velocity-position fluctuation covariance can be obtained by multiplying *x *- <*x *> and *v *- <*v *>, and taking the ensemble average. The *K*(*b*,*q*,*t*_1_) in Equation 14 is

(37) $$\begin{array}{c} K\left(b,q,t_{1}\right)\\ \;\;=\int_{0}^{t_{1}}\left[s\left(-\frac{b^{2}}{4},q,u\right)c\left(-\frac{b^{2}}{4},q,t_{1}\right)-c\left(-\frac{b^{2}}{4},q,u\right)s\left(-\frac{b^{2}}{4},q,t_{1}\right)\right]\times \\ \;\;\;\;\left[s\left(-\frac{b^{2}}{4},q,u\right)c^{\prime }\left(-\frac{b^{2}}{4},q,t_{1}\right)-c\left(-\frac{b^{2}}{4},q,u\right)s^{\prime }\left(-\frac{b^{2}}{4},q,t_{1}\right)\right]e^{b\left(u-t_{1}\right)}du\\ \;\;\;-\left(\frac{b}{2}\right)\int_{0}^{t_{1}}{\left[s\left(-\frac{b^{2}}{4},q,u\right)c\left(-\frac{b^{2}}{4},q,t_{1}\right)-c\left(-\frac{b^{2}}{4},q,u\right)s\left(-\frac{b^{2}}{4},q,t_{1}\right)\right]}^{2}e^{b\left(u-t_{1}\right)}du \end{array}$$

and it can be written in form:

(38) $$K\left(b,q,t_{1}\right)=J_{2}\left(b,q,t_{1}\right)-\frac{b}{2}J_{3}\left(b,q,t_{1}\right)$$

where *J*_2_(*b*,*q*,*t*_1_), and *J*_3_(*b*,*q*,*t*_1_) are defined in Appendix 2.

## Supplementary Material

Additional file 1 **Supplementary information**. SI 1, detailed derivation of Equation 8; SI 2, Mathieu exponents for various *b *and *q*; Figure S1, variation of Mathieu exponent with *q *for a given *b*; SI 3, derivation of Equation 9; SI 4, time histories of *σ*_xx_, *σ*_vv_, and *σ*_xv_; Figure S2, time histories of *σ*_xx_, *σ*_vv_, and *σ*_xv _for *b *= 2.0. Click here for file
