# Supplementary material for: Thermal noise in aqueous quadrupole micro- and nano-traps
Source: Nanoscale Res Lett. 2012 Feb 27;7(1):156. doi: 10.1186/1556-276X-7-156 (PMC3382431; doi:10.1186/1556-276X-7-156)
Supplement: Additional file 1 — Supplementary information. SI 1, detailed derivation of Equation 8; SI 2, Mathieu exponents for various b and q; Figure S1, variation of Mathieu exponent with q for a given b; SI 3, derivation of Equation 9; SI 4, time histories of σxx, σvv, and σxv; Figure S2, time histories of σxx, σvv, and σxv for b = 2.0. [file 1556-276X-7-156-S1.PDF]

Supplementary Information for "Thermal Noises in an Aqueous Quadrupole Micro- and Nano-Trap"

Jae Hyun Park and Predrag S. Krstić[1]*

Physics Division, Oak Ridge National Laboratory, Oak Ridge, TN 37831

E-mail: krsticp@ornl.gov

*corresponding author,
Dr. Predrag S. Krstić
Physics Division
Oak Ridge National Laboratory
PO Box 2008, Bldg. 6010
Oak Ridge, TN 37831-6372
Tel. (865) 574-4701
Fax (865) 574-1118
E-mail:krsticp@ornl.gov

Table of Contents

SI 1. Detailed Derivation of Equation (9)
SI 2. Mathieu exponents for various b and q
SI 3. Derivation of Equations (11)
SI 4. Time Histories of σ_{xx}, σ_{vv}, and σ_{xv}

SI 1. Detailed Derivation of Equation (9)

The integration I(b,q,t_1) in equation (8) is expressed by

$$I(b,q,t_1)=\int_0^{t_1}\left[s\left(-\frac{1}{4}b^2,q,u\right)c\left(-\frac{1}{4}b^2,q,t_1\right)-c\left(-\frac{1}{4}b^2,q,u\right)s\left(-\frac{1}{4}b^2,q,t_1\right)\right]^2 e^{b(u-t_1)}du \quad \text{(S1)}$$

The Mathieu cosine and sine functions are related with the Floquet solution of Mathieu equation, $F(a,q,t)$ as

$$c(a,q,t)=\frac{F(a,q,t)+F(a,q,-t)}{2F(a,q,0)}, \quad \text{(S2a)}$$

$$s(a,q,t)=\frac{F(a,q,t)-F(a,q,-t)}{2F'(a,q,0)}. \quad \text{(S2b)}$$

Hereafter, for brevity $a=-b^2/4$ unless specified otherwise. Since the Floquet solution of Mathieu equation, $F(a,q,t)$, has the form of

$$F(a,q,t)=\exp(i\mu t)P(a,q,t). \quad \text{(S3)}$$

where μ is Mathieu exponent (complex number) and $P(a,q,t)$ is the complex valued function which is *periodic* with period π. From equations (S1) and (S2), $c(a,q,t)$ and $s(a,q,t)$ can be expressed as:

$$c(a,q,t)=\frac{\exp(i\mu t)P(a,q,t)+\exp(-i\mu t)P(a,q,-t)}{2F(a,q,0)}, \quad \text{(S4a)}$$

$$s(a,q,t)=\frac{\exp(i\mu t)P(a,q,t)-\exp(-i\mu t)P(a,q,-t)}{2F'(a,q,0)}. \quad \text{(S4b)}$$

By inserting equations (S3) into equation (S1), then we obtain

$$\left(4\left[F(a,q,0)F'(a,q,0)\right]^2\right)I(a,q,t_1)=$$
$$\int_0^{t_1}\exp\left[(b+i2\mu)(u-t)\right]\left[P(a,q,u)P(a,q,-t_1)\right]^2du$$
$$-2\int_0^{t_1}\exp\left[b(u-t)\right]\left[P(a,q,u)P(a,q,-u)P(a,q,t_1)P(a,q,-t_1)\right]du$$
$$+\int_0^{t_1}\exp\left[(b-i2\mu)(u-t)\right]\left[P(a,q,-u)P(a,q,t_1)\right]^2du$$
$$=\tilde{I}_1(a,q,t_1)-2\tilde{I}_2(a,q,t_1)+\tilde{I}_3(a,q,t_1) \quad . \qquad \text{(S5)}$$

Rewriting $t_1=n\pi+\tau$ ($0\le\tau<\pi$, n is non-zero integer) and using the division property of integration,

$$\int_0^{t_1}=\int_0^{\pi}+\int_{\pi}^{2\pi}+\cdots+\int_{(n-2)\pi}^{(n-1)\pi}+\int_{(n-1)\pi}^{n\pi}+\int_{n\pi}^{n\pi+\tau} \quad , \qquad \text{(S6)}$$

and the π periodicity of $P(a,q,t)$, *i.e.* $P(a,q,t)=P(a,q,n\pi+t)$, $\tilde{I}_1(a,q,t_1)$, $\tilde{I}_2(a,q,t_1)$, $\tilde{I}_3(a,q,t_1)$ in equation (B4) can be computed as

$$\tilde{I}_1(a,q,t_1)$$
$$=\sum_{l=1}^{n}e^{(b+i2\mu)\pi(l-1)}\int_0^{\pi}e^{(b+i2\mu)(u-n\pi-\tau)}\left[P(a,q,u)P(a,q,-\tau)\right]^2du$$
$$+\int_0^{\tau}e^{(b+i2\mu)(u-\tau)}\left[P(a,q,u)P(a,q,-\tau)\right]^2du$$
$$=\left(\frac{1-e^{-(b+i2\mu)n\pi}}{e^{(b+i2\mu)\pi}-1}\right)\int_0^{\pi}e^{(b+i2\mu)(u-\tau)}\left[P(a,q,u)P(a,q,-\tau)\right]^2du$$
$$+\int_0^{\tau}e^{(b+i2\mu)(u-\tau)}\left[P(a,q,u)P(a,q,-\tau)\right]^2du \quad , \qquad \text{(S7a)}$$

$$\tilde{I}_2(a,q,t_1)$$
$$=\sum_{l=1}^{n}e^{b\pi(l-1)}\int_0^{\pi}e^{b(u-n\pi-\tau)}\left[P(a,q,u)P(a,q,-u)P(a,q,\tau)P(a,q,-\tau)\right]du$$
$$+\int_0^{\tau}e^{b(u-\tau)}\left[P(a,q,u)P(a,q,-u)P(a,q,\tau)P(a,q,-\tau)\right]du$$
$$=\left(\frac{1-e^{-nb\pi}}{e^{b\pi}-1}\right)\int_0^{\pi}e^{b(u-\tau)}\left[P(a,q,u)P(a,q,-u)P(a,q,\tau)P(a,q,-\tau)\right]du$$
$$+\int_0^{\tau}e^{b(u-\tau)}\left[P(a,q,u)P(a,q,-u)P(a,q,\tau)P(a,q,-\tau)\right]dv \quad , \qquad \text{(S7b)}$$

$$\begin{aligned}
&\tilde{I}_3(a,q,t_1) \\
&= \sum_{l=1}^{n} e^{(b-i2\mu)\pi(l-1)} \int_0^{\pi} e^{(b-i2\mu)(u-n\pi-\tau)} \left[P(a,q,u)P(a,q,-\tau)\right]^2 du \\
&\quad + \int_0^{\tau} e^{(b-i2\mu)(u-\tau)} \left[P(a,q,u)P(a,q,-\tau)\right]^2 du \\
&= \left(\frac{1-e^{-(b-i2\mu)n\pi}}{e^{(b+i2\mu)\pi}-1}\right) \int_0^{\pi} e^{(b-i2\mu)(u-\tau)} \left[P(a,q,u)P(a,q,-\tau)\right]^2 du \\
&\quad + \int_0^{\tau} e^{(b-i2\mu)(v-\tau)} \left[P(a,q,v)P(a,q,-\tau)\right]^2 dv
\end{aligned} \quad . \qquad \text{(S7c)}$$

Thereby, $I(a,q,t_1)$ can be written as:

$$I(a,q,t_1) = I_1(a,q,\tau) + \frac{1}{4}\left[I_{2a}^{n}(a,q,\tau) - 2I_{2b}^{n}(a,q,\tau) + I_{2c}^{n}(a,q,\tau)\right], \qquad \text{(S8a)}$$

$$I_1(a,q,\tau) = \int_0^{\tau} \left[s(a,q,u)c(a,q,\tau) - c(a,q,u)s(a,q,\tau)\right]^2 e^{b(u-\tau)} du \quad , \qquad \text{(S8b)}$$

$$I_{2a}^{n}(a,q,\tau) = \left(\frac{1-e^{-(b+i2\mu)n\pi}}{e^{(b+i2\mu)\pi}-1}\right) \int_0^{\pi} e^{b(u-\tau)} \left[\frac{F(a,q,u)F(a,q,-\tau)}{F(a,q,0)F'(a,q,0)}\right]^2 du \quad , \qquad \text{(S8c)}$$

$$I_{2b}^{n}(b,q,\tau) = \left(\frac{1-e^{-nb\pi}}{e^{b\pi}-1}\right) \int_0^{\pi} e^{b(u-\tau)} \frac{F(a,q,u)F(a,q,-u)F(a,q,\tau)F(a,q,-\tau)}{\left[F(a,q,0)F'(a,q,0)\right]^2} du \quad , \qquad \text{(S8d)}$$

$$I_{2c}^{n}(b,q,\tau) = \left(\frac{1-e^{-(b-i2\mu)n\pi}}{e^{(b-i2\mu)\pi}-1}\right) \int_0^{\pi} e^{b(u-\tau)} \left[\frac{F(a,q,-u)F(a,q,\tau)}{F(a,q,0)F'(a,q,0)}\right]^2 du \quad . \qquad \text{(S8e)}$$

The equations (S8b-e) present the integrations $I_1(b,q,\tau)$, $I_{2a}^{n}(b,q,\tau)$, $I_{2b}^{n}(b,q,\tau)$, and $I_{2c}^{n}(b,q,\tau)$ in equation (9).

In order to achieve the stable long-time behavior of $I(a,q,t_1)$, all the expressions of equations (S8c)-(S8e) need to converge and the exponential term $e^{-(b-i2\mu)n\pi}$ should vanish for $n\to\infty$. The necessary condition for this is $\beta < b/2$ where β is the imaginary part of Mathieu exponent, $\mu=\alpha+i\beta$. It is same as the condition that the mean motion of particle is stable [S1].

The long-time behaviors of I_{2a}, I_{2b}, and I_{2c} in stable condition can be obtained with $n\to\infty$,

$$I_{2a}^{\infty}(a,q,\tau)=\left(\frac{1}{e^{(b+i2\mu)\pi}-1}\right)\int_0^{\pi}e^{b(u-\tau)}\left[\frac{F(a,q,u)F(a,q,-\tau)}{F(a,q,0)F'(a,q,0)}\right]^2 du \quad , \tag{S9a}$$

$$I_{2b}^{\infty}(a,q,\tau)=\left(\frac{1}{e^{b\pi}-1}\right)\int_0^{\pi}e^{b(u-\tau)}\frac{F(a,q,u)F(a,q,-u)F(a,q,\tau)F(a,q,-\tau)}{\left[F(a,q,0)F'(a,q,0)\right]^2}du \quad , \tag{S9b}$$

$$I_{2c}^{\infty}(a,q,\tau)=\left(\frac{1}{e^{(b-i2\mu)\pi}-1}\right)\int_0^{\pi}e^{b(u-\tau)}\left[\frac{F(a,q,-u)F(a,q,\tau)}{F(a,q,0)F'(a,q,0)}\right]^2 du \quad , \tag{S9c}$$

SI 2. Mathieu Exponents for Various b and q

In the aid of figure S1, we can compute the q-range satisfying the condition of $0<\beta<b/2$ for q given b.

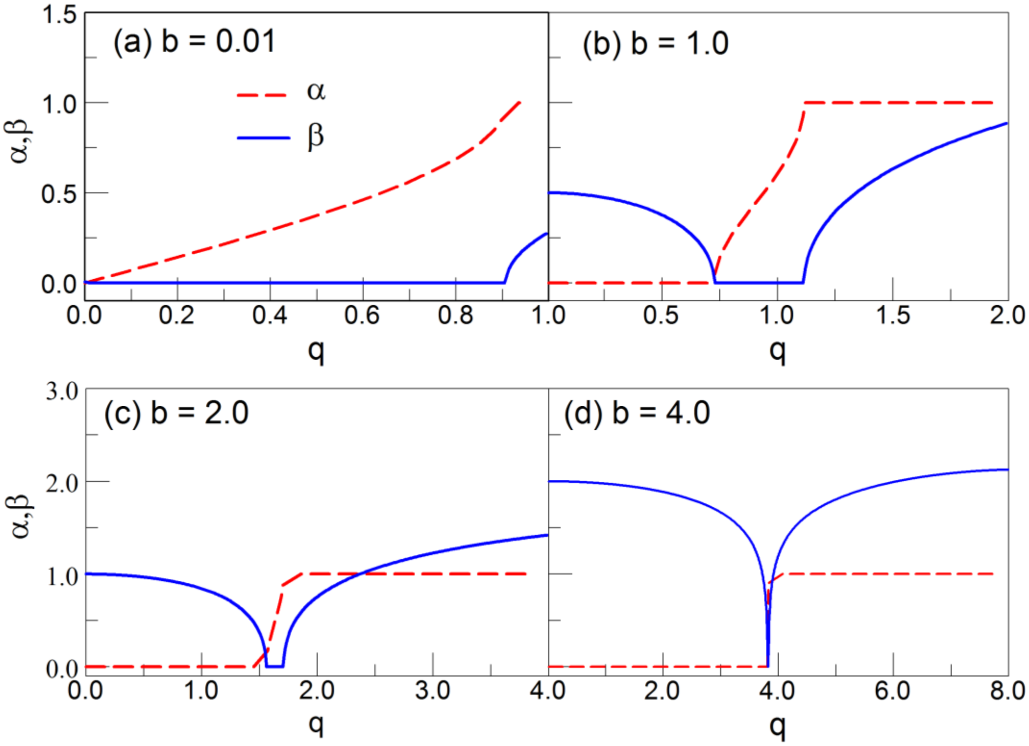

Figure S1. Variation of Mathieu exponent with q for a given b. α and β are the real and imaginary parts of Mathieu exponent μ, respectively.

SI 3. Derivation of Equations (11)

The integration J(b,q,t$_1$) in equation (10) is originally given by

$$\begin{aligned} J\left(b,q,t_1\right) = \int_0^{t_1} \Bigg[& s\left(-\frac{1}{4}b^2,q,u\right)c'\left(-\frac{1}{4}b^2,q,t_1\right) - \frac{b}{2}s\left(-\frac{1}{4}b^2,q,u\right)c\left(-\frac{1}{4}b^2,q,t_1\right) \\ & -c\left(-\frac{1}{4}b^2,q,u\right)s'\left(-\frac{1}{4}b^2,q,t_1\right) + \frac{b}{2}c\left(-\frac{1}{4}b^2,q,u\right)s\left(-\frac{1}{4}b^2,q,t_1\right)\Bigg]^2 e^{b(u-t_1)}du \end{aligned} . \quad \text{(S10)}$$

The integration of J(b,q,t$_1$), equation (S10), can be re-written as the summation of three terms:

$$J\left(a,q,t_1\right) = J_1\left(a,q,t_1\right) - bJ_2\left(a,q,t_1\right) + \frac{b^2}{4}J_3\left(a,q,t_1\right). \quad \text{(S11a)}$$

with

$$J_1\left(a,q,t_1\right) = \int_0^{t_1}\left[s\left(a,q,u\right)c'\left(a,q,t_1\right) - c\left(a,q,u\right)s'\left(a,q,t_1\right)\right]^2 e^{b(u-t_1)}du . \quad \text{(S11b)}$$

$$\begin{aligned} J_2\left(a,q,t_1\right) = \int_0^{t_1} \Big\{ & \left[s\left(a,q,u\right)c'\left(a,q,t_1\right) - c\left(a,q,u\right)s'\left(a,q,t_1\right)\right] \\ & \times\left[s\left(a,q,u\right)c\left(a,q,t_1\right) - c\left(a,q,u\right)s\left(a,q,t_1\right)\right]\Big\} e^{b(u-t_1)}du \end{aligned} . \quad \text{(S11c)}$$

$$\begin{aligned} J_3\left(a,q,t_1\right) &= \int_0^{t_1}\left[s\left(a,q,u\right)c\left(a,q,t_1\right) - c\left(a,q,u\right)s\left(a,q,t_1\right)\right]^2 e^{b(u-t_1)}du \\ &= I_{exact}\left(a,q,t_1\right) \end{aligned} . \quad \text{(S11d)}$$

Here $a = -b^2/4$. Interestingly, J$_3$(b,q,t$_1$) is equal to I(b,q,t$_1$) in the fluctuation of position. Following the same procedure for the fluctuation of positions in S1, we can write the time t$_1$as t$_1$ = nπ+τ (0≤τ<π, n is non-zero integer) and then, the each term can be written as follow:

For J$_1$(b,q,t$_1$),

$$J_1\left(a,q,t_1\right) = J_{11}\left(a,q,\tau\right) + \frac{1}{4}\left[J_{12a}^n\left(a,q,\tau\right) + 2J_{12b}^n\left(a,q,\tau\right) + J_{12c}^n\left(a,q,\tau\right)\right], \quad \text{(S12a)}$$

with

$$J_{11}\left(a,q,\tau\right) = \int_0^{\tau}\left[s\left(a,q,u\right)c'\left(a,q,\tau\right) - c\left(a,q,u\right)s'\left(a,q,\tau\right)\right]^2 e^{b(u-\tau)}du , \quad \text{(S12b)}$$

$$J_{12a}^n\left(a,q,\tau\right) = \frac{\left(1-e^{-(b+i2\mu)n\pi}\right)}{e^{(b+i2\mu)\pi}-1}\int_0^{\pi} e^{b(v-\tau)}\left[\frac{F\left(a,q,u\right)F'\left(a,q,-\tau\right)}{F\left(a,q,0\right)F'\left(a,q,0\right)}\right]^2 du , \quad \text{(S12c)}$$

$$J_{12b}^{n}\left(a,q,\tau\right)=\frac{\left(1-e^{-nb\pi}\right)}{e^{b\pi}-1}\int_0^{\pi}e^{b(v-\tau)}\frac{F\left(a,q,u\right)F'\left(a,q,-\tau\right)F\left(a,q,\tau\right)F'\left(a,q,-u\right)}{\left[F\left(a,q,0\right)F'\left(a,q,0\right)\right]^2}du\,, \qquad \text{(S12d)}$$

$$J_{12c}^{n}\left(a,q,\tau\right)=\frac{\left(1-e^{-(b-i2\mu)n\pi}\right)}{e^{(b+i2\mu)\pi}-1}\int_0^{\pi}e^{b(v-\tau)}\left[\frac{F\left(a,q,-u\right)F'\left(a,q,\tau\right)}{F\left(a,q,0\right)F'\left(a,q,0\right)}\right]^2du\,. \qquad \text{(S12e)}$$

For $J_2(b,q,t_1)$,

$$J_2\left(a,q,t_1\right)=J_{21}\left(a,q,\tau\right)+\frac{1}{4}\left[J_{22a}^{n}\left(a,q,\tau\right)+J_{22b}^{n}\left(a,q,\tau\right)-J_{22c}^{n}\left(a,q,\tau\right)\right], \qquad \text{(S13a)}$$

with

$$\begin{aligned}J_{21}\left(a,q,\tau\right)=\int_0^{\tau}&\left\{\left[s\left(a,q,u\right)c'\left(a,q,\tau\right)-c\left(a,q,u\right)s'\left(a,q,\tau\right)\right]\right.\\&\left.\times\left[s\left(a,q,u\right)c\left(a,q,\tau\right)-c\left(a,q,u\right)s\left(a,q,\tau\right)\right]\right\}e^{b(u-\tau)}du\end{aligned}\,, \qquad \text{(S13b)}$$

$$J_{22a}^{n}\left(a,q,\tau\right)=\left(\frac{1-e^{-(b+i2\mu)n\pi}}{e^{(b+i2\mu)\pi}-1}\right)\int_0^{\pi}e^{b(v-\tau)}\frac{\left[F\left(a,q,u\right)\right]^2F\left(a,q,-\tau\right)F'\left(a,q,-\tau\right)}{F\left(a,q,0\right)F'\left(a,q,0\right)}du\,, \qquad \text{(S13c)}$$

$$\begin{aligned}J_{22b}^{n}\left(a,q,\tau\right)=&\left(\frac{1-e^{-nb\pi}}{e^{b\pi}-1}\right)\\&\times\int_0^{\pi}e^{b(u-\tau)}\frac{F\left(a,q,u\right)F\left(a,q,-u\right)\left[F'\left(a,q,\tau\right)F\left(a,q,-\tau\right)+F\left(a,q,\tau\right)F'\left(a,q,-\tau\right)\right]}{\left[F\left(a,q,0\right)F'\left(a,q,0\right)\right]^2}du\end{aligned}\,, \qquad \text{(S13d)}$$

$$J_{22c}^{n}\left(a,q,\tau\right)=\left(\frac{1-e^{-(b-i2\mu)n\pi}}{e^{(b+i2\mu)\pi}-1}\right)\int_0^{\pi}e^{b(v-\tau)}\frac{\left[F\left(a,q,-u\right)\right]^2F\left(a,q,\tau\right)F'\left(a,q,\tau\right)}{\left[F\left(a,q,0\right)F'\left(a,q,0\right)\right]^2}du\,. \qquad \text{(S13e)}$$

For $J_3(b,q,t_1)$,

$$J_3\left(a,q,t_1\right)=J_{31}\left(a,q,\tau\right)+\frac{1}{4}\left[J_{32a}^{n}\left(a,q,\tau\right)-2J_{32b}^{n}\left(a,q,\tau\right)+J_{32c}^{n}\left(a,q,\tau\right)\right], \qquad \text{(S14a)}$$

with

$$J_{31}\left(a,q,\tau\right)=\int_0^{\tau}\left[s\left(a,q,u\right)c\left(a,q,\tau\right)-c\left(a,q,u\right)s\left(a,q,\tau\right)\right]^2e^{b(u-\tau)}du\,, \qquad \text{(S14b)}$$

$$J_{32a}^{n}(a,q,\tau)=\left(\frac{1-e^{-(b+i2\mu)n\pi}}{e^{(b+i2\mu)\pi}-1}\right)\int_0^{\pi}e^{b(u-\tau)}\left[\frac{F(a,q,u)F(a,q,-\tau)}{F(a,q,0)F'(a,q,0)}\right]^2 du\,, \qquad \text{(S14c)}$$

$$J_{32b}^{n}(a,q,\tau)=\left(\frac{1-e^{-nb\pi}}{e^{b\pi}-1}\right)\int_0^{\pi}e^{b(u-\tau)}\frac{F(a,q,u)F(a,q,-u)F(a,q,\tau)F(a,q,-\tau)}{\left[F(a,q,0)F'(a,q,0)\right]^2}du\,, \qquad \text{(S14d)}$$

$$J_{32c}^{n}(a,q,\tau)=\left(\frac{1-e^{-(b-i2\mu)n\pi}}{e^{(b-i2\mu)\pi}-1}\right)\int_0^{\pi}e^{b(u-\tau)}\left[\frac{F(a,q,-u)F(a,q,\tau)}{F(a,q,0)F'(a,q,0)}\right]^2 du\,. \qquad \text{(S14e)}$$

Collecting all the expressions in above, equation (S11a) can be written in the following simple forms as below:

$$J(a,q,t_1)=J_1(a,q,t_1)-bJ_2(a,q,t_1)+\frac{b^2}{4}J_3(a,q,t_1)\,, \qquad \text{(S11a)}$$

For $J_1(b,q,t_1)$,

$$J_1(b,q,t_1)=J_{11}(b,q,\tau)+\frac{1}{4}\left[J_{12a}^{n}(b,q,\tau)+2J_{12b}^{n}(b,q,\tau)+J_{12c}^{n}(b,q,\tau)\right], \qquad \text{(S15a)}$$

with

$$J_{12a}^{n}(b,q,\tau)=J_{12a}^{\infty}(b,q,\tau)\cdot\left\{1-\left[e^{-(b+i2\mu)\pi}\right]^n\right\}, \qquad \text{(S15b)}$$

$$J_{12b}^{n}(b,q,\tau)=J_{12b}^{\infty}(b,q,\tau)\cdot\left\{1-\left[e^{-b\pi}\right]^n\right\}, \qquad \text{(S15c)}$$

$$J_{12c}^{n}(b,q,\tau)=J_{12c}^{\infty}(b,q,\tau)\cdot\left\{1-\left[e^{-(b-i2\mu)\pi}\right]^n\right\}, \qquad \text{(S15d)}$$

$$J_{11}(a,q,\tau)=\int_0^{\tau}\left[s(a,q,u)c'(a,q,\tau)-c(a,q,u)s'(a,q,\tau)\right]^2 e^{b(u-\tau)}du\,, \qquad \text{(S15e)}$$

$$J_{22a}^{\infty}(a,q,\tau)=\left(\frac{1}{e^{(b+i2\mu)\pi}-1}\right)\int_0^{\pi}e^{b(u-\tau)}\left[\frac{F(a,q,u)F'(a,q,-\tau)}{F(a,q,0)F'(a,q,0)}\right]^2 du\,, \qquad \text{(S15f)}$$

$$J_{22a}^{\infty}(a,q,\tau)=\left(\frac{1}{e^{b\pi}-1}\right)\int_0^{\pi}e^{b(u-\tau)}\frac{F(a,q,u)F(a,q,-u)F'(a,q,\tau)F'(a,q,-\tau)}{\left[F(a,q,0)F'(a,q,0)\right]^2}du\,, \qquad \text{(S15g)}$$

and

$$J_{22a}^{\infty}\left(a,q,\tau\right)=\left(\frac{1}{e^{\left(b-i2\mu\right)\pi}-1}\right)\int_{0}^{\pi}e^{b\left(u-\tau\right)}\left[\frac{F\left(a,q,-u\right)F'\left(a,q,\tau\right)}{F\left(a,q,0\right)F'\left(a,q,0\right)}\right]^{2}du\,. \qquad \text{(S15h)}$$

For $J_2(b,q,t_1)$,

$$J_{2}\left(b,q,t_{1}\right)=J_{21}\left(b,q,\tau\right)-\frac{1}{4}\left[J_{22a}^{n}\left(b,q,\tau\right)+J_{22b}^{n}\left(b,q,\tau\right)-J_{22c}^{n}\left(b,q,\tau\right)\right], \qquad \text{(S16a)}$$

with

$$J_{22a}^{n}\left(b,q,\tau\right)=J_{22a}^{\infty}\left(b,q,\tau\right)\cdot\left\{1-\left[e^{-\left(b+i2\mu\right)\pi}\right]^{n}\right\}, \qquad \text{(S16b)}$$

$$J_{22b}^{n}\left(b,q,\tau\right)=J_{22b}^{\infty}\left(b,q,\tau\right)\cdot\left\{1-\left[e^{-b\pi}\right]^{n}\right\}, \qquad \text{(S16c)}$$

$$J_{22c}^{n}\left(b,q,\tau\right)=J_{22c}^{\infty}\left(b,q,\tau\right)\cdot\left\{1-\left[e^{-\left(b-i2\mu\right)\pi}\right]^{n}\right\}, \qquad \text{(S16d)}$$

$$\begin{aligned}J_{21}\left(a,q,\tau\right)=\int_{0}^{\tau}&\left[s\left(a,q,u\right)c'\left(a,q,\tau\right)-c\left(a,q,u\right)s'\left(a,q,\tau\right)\right]\\&\times\left[s\left(a,q,u\right)c\left(a,q,\tau\right)-c\left(a,q,u\right)s\left(a,q,\tau\right)\right]e^{b\left(u-\tau\right)}du\end{aligned}, \qquad \text{(S16e)}$$

$$J_{22a}^{\infty}\left(a,q,\tau\right)=\left(\frac{1}{e^{\left(b+i2\mu\right)\pi}-1}\right)\int_{0}^{\pi}e^{b\left(v-\tau\right)}\frac{\left[F\left(a,q,v\right)\right]^{2}F\left(a,q,-\tau\right)F'\left(a,q,-\tau\right)}{\left[F\left(a,q,0\right)F'\left(a,q,0\right)\right]^{2}}dv, \qquad \text{(S16f)}$$

$$J_{22b}^{\infty}\left(a,q,\tau\right)=\left(\frac{1}{e^{b\pi}-1}\right)\int_{0}^{\pi}e^{b\left(v-\tau\right)}\frac{F\left(a,q,v\right)F\left(a,q,-v\right)\left[F'\left(a,q,\tau\right)F\left(a,q,-\tau\right)-F\left(a,q,\tau\right)F'\left(a,q,-\tau\right)\right]}{\left[F\left(a,q,0\right)F'\left(a,q,0\right)\right]^{2}}dv, \qquad \text{(S16g)}$$

and

$$J_{22c}^{\infty}\left(a,q,\tau\right)=\left(\frac{1}{e^{\left(b-i2\mu\right)\pi}-1}\right)\int_{0}^{\pi}e^{b\left(v-\tau\right)}\frac{\left[F\left(a,q,v\right)\right]^{2}F\left(a,q,-\tau\right)F'\left(a,q,-\tau\right)}{\left[F\left(a,q,0\right)F'\left(a,q,0\right)\right]^{2}}dv\,. \qquad \text{(S16h)}$$

For $J_3(b,q,t_1)$,

$$J_{3}\left(b,q,t_{1}\right)=J_{31}\left(b,q,\tau\right)+\frac{1}{4}\left[J_{32a}^{n}\left(b,q,\tau\right)-2J_{32b}^{n}\left(b,q,\tau\right)-J_{32c}^{n}\left(b,q,\tau\right)\right], \qquad \text{(S17a)}$$

with

$$J_{32a}^{n}(b,q,\tau) = J_{32a}^{\infty}(a,q,\tau)\cdot\left\{1-\left[e^{-(b+i2\mu)\pi}\right]^{n}\right\}, \tag{S17b}$$

$$J_{32b}^{n}(b,q,\tau) = J_{32b}^{\infty}(a,q,\tau)\cdot\left\{1-\left[e^{-b\pi}\right]^{n}\right\}, \tag{S17c}$$

$$J_{32c}^{n}(b,q,\tau) = J_{32c}^{\infty}(a,q,\tau)\cdot\left\{1-\left[e^{-(b-i2\mu)\pi}\right]^{n}\right\}, \tag{S17d}$$

$$J_{31}(a,q,\tau) = \int_0^{\tau}\left[s(a,q,u)c(a,q,\tau)-c(a,q,u)s(a,q,\tau)\right]^2 e^{b(u-\tau)}du\ , \tag{S17e}$$

$$J_{32a}^{\infty}(a,q,\tau) = \left(\frac{1}{e^{(b+i2\mu)\pi}-1}\right)\int_0^{\pi} e^{b(u-\tau)}\left[\frac{F(a,q,u)F(a,q,-\tau)}{F(a,q,0)F'(a,q,0)}\right]^2 du\ , \tag{S17f}$$

$$J_{32b}^{\infty}(a,q,\tau) = \left(\frac{1}{e^{b\pi}-1}\right)\int_0^{\pi} e^{b(u-\tau)}\frac{F(a,q,u)F(a,q,-u)F(a,q,\tau)F(a,q,-\tau)}{\left[F(a,q,0)F'(a,q,0)\right]^2}du\, , \tag{S17g}$$

and

$$J_{32c}^{\infty}(a,q,\tau) = \left(\frac{1}{e^{(b-i2\mu)\pi}-1}\right)\int_0^{\pi} e^{b(u-\tau)}\left[\frac{F(a,q,-u)F(a,q,\tau)}{F(a,q,0)F'(a,q,0)}\right]^2 du\ . \tag{S17h}$$

SI 4. Time Histories of σ_{xx}, σ_{vv}, and σ_{xv} for large b = 4.0

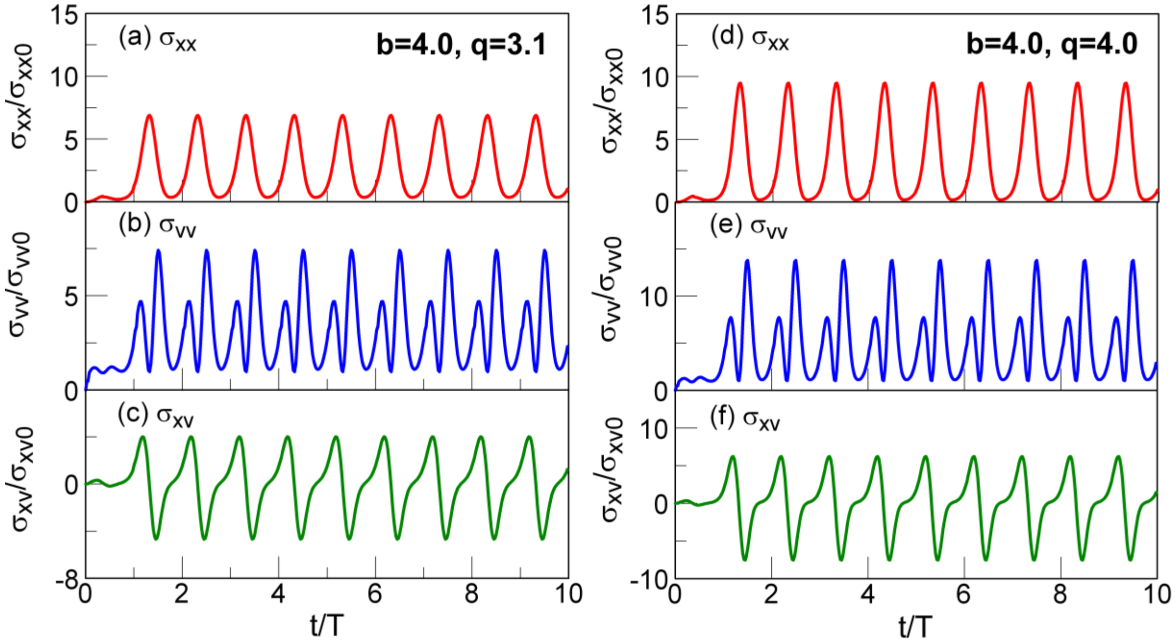

Figure S2. Time histories of σ_{xx}, σ_{vv}, and σ_{xv} for b=2.0: (a), (b), (c) for q = 3.1; (d), (e), (f) for q =4.0.

Figures S2 show the transient behaviour of σ_{xx}, σ_{vv}, and σ_{xv} for b=4.0 and q = 3.1 (a, b, c) and for b=4.0 and q = 4.0 (d, e, f). If q further increases (q > 4), the position fluctuation σ_{xx} is diverging along with σ_{vv}, and σ_{xv} and the fourth frequency mode starts to contribute in addition to the first three modes, which dominates for q=3.1. It should be noted that the velocity fluctuations are increased more than position and covariance fluctuations, which indicates that the divergence of the position fluctuation originates from the velocity and propagates to the position divergence with the aid of covariance.
